# Age-related gene expression in luminal epithelial cells is driven by a microenvironment made from myoepithelial cells

**DOI:** 10.18632/aging.101298

**Published:** 2017-10-09

**Authors:** Masaru Miyano, Rosalyn W. Sayaman, Marcus H. Stoiber, Chun-Han Lin, Martha R. Stampfer, James B. Brown, Mark A. LaBarge

**Affiliations:** ^1^ Department of Population Sciences, City of Hope, Duarte, CA 91010, USA; ^2^ Center for Cancer and Aging, City of Hope, Duarte, CA 91010 USA; ^3^ Biological Systems and Engineering Division, Lawrence Berkeley National Laboratory, Berkeley, CA 94720, USA; ^4^ Envrionmental Genomics and Systems Biology Division, Lawrence Berkeley National Laboratory, Berkeley, CA 94720, USA; ^5^ Center for Cancer Biomarkers Research, University of Bergen, Bergen, Norway; ^6^ Department of Environmental Bioinformatics, University of Birmingham, Birmingham, UK

**Keywords:** mammary epithelia, breast cancer, aging, microenvironment, epigenetic

## Abstract

Luminal epithelial cells in the breast gradually alter gene and protein expression with age, appearing to lose lineage-specificity by acquiring myoepithelial-like characteristics. We hypothesize that the luminal lineage is particularly sensitive to microenvironment changes, and age-related microenvironment changes cause altered luminal cell phenotypes. To evaluate the effects of different microenvironments on the fidelity of epigenetically regulated luminal and myoepithelial gene expression, we generated a set of lineage-specific probes for genes that are controlled through DNA methylation. Culturing primary luminal cells under conditions that favor myoepithelial propogation led to their reprogramming at the level of gene methylation, and to a more myoepithelial-like expression profile. Primary luminal cells’ lineage-specific gene expression could be maintained when they were cultured as bilayers with primary myoepithelial cells. Isogenic stromal fibroblast co-cultures were unable to maintain the luminal phenotype. Mixed-age luminal-myoepithelial bilayers revealed that luminal cells adopt transcription and methylation patterns consistent with the chronological age of the myoepithelial cells. We provide evidence that the luminal epithelial phenotype is exquisitely sensitive to microenvironment conditions, and that states of aging are cell non-autonomously communicated through microenvironment cues over at least one cell diameter.

## INTRODUCTION

The incidence of nearly all carcinomas increases with age; more than 80% of breast cancers are diagnosed in women over 50, but the biology underlying this striking consequence of aging is not understood. The mammary gland is an arbor-like structure with bilayered ductal epithelia containing an inner layer of secretory luminal epithelial cells (LEP), surrounded by an outer layer of contractile and tumor suppressive myoepithelial cells (MEP) [1]. Aging in the breast stroma is characterized by prominent microenvironment changes such as increased fat content and decreased connective tissue [2, 3]. In the epithelia, aging is accompanied by increases in estrogen receptor expression [4], decreased proportions of tumor suppressive MEP, accumulation of dysfunctional multipotent progenitors, and acquisition by LEP of biochemical and molecular properties normally observed only in MEP of younger women (e.g. expression of keratin 14, YAP, TAZ, and integrin alpha 6, and decreased expression of keratin 19) [5, 6]. The cell-of-origin for most breast cancers is thought to reside in the luminal or supra-basal regions directly adjacent to the apical surfaces of the MEP. Thus, an understanding of how LEP-MEP interactions may regulate LEP lineage fidelity, and its loss and dysregulation with age, may elucidate the mechanism behind increased breast cancer susceptibility with age.

Aging-associated phenotypes in a number of tissues have been correlated with transcriptional and epigenetic changes [7-9], and altered DNA methylation patterns provide a reasonable explanation for the stability of age-related phenotypes [10-12]. Indeed, the chronological age of normal human mammary epithelial cells (HMEC) influences the transformed subtype of HMEC when immortalized in vitro, with cells from older women yielding a more luminal phenotype [13]. Thus, age-related epigenetic states may be important in establishing breast cancer subtypes. On the other hand, changes to the microenvironment have been shown to impose distinct gene expression patterns [14, 15], direct lineage specification [16] and to contribute to age-related gene expression changes. For example, in the skeletal muscle context, it was shown with transplantation and parabiosis experiments that the microenvironment strongly influenced the functional age of that tissue [17, 18].

Here we test the hypothesis that the microenvironment is a crucial determinant of LEP lineage specificity, and that age-specific changes of the microenvironment drive age-related phenotypes in LEP. LEP-lineage fidelity was examined in engineered microenvironments built from a small number of cells isolated from different individuals, and assayed using a set of quantitative (q)PCR probes developed for a subset of LEP and MEP specific genes that were controlled by promoter DNA methyl-tion. We demonstrate that culturing primary LEP in conditions that favor MEP propagation leads to a down-regulation of a number of LEP-specific genes that is associated with increased promoter methylation, and an upregulation of MEP genes. Mixed-age, multi-lineage co-cultures demonstrate that the cellular micro-environment, specifically the apical surface of MEP on which LEP reside, can impose age- and lineage-specific gene expression patterns, and we provide evidence that concordant changes in DNA methylation occur. Thus we show that aging states in breast epithelia are communicat-ed via microenvironment cues by precise cell-cell contact between the LEP and MEP, but not by cells one layer away – e.g. stromal fibroblasts. The LEP phenotype is exquisitely sensitive to microenvironment conditions.

## RESULTS

### The relationship between promoter DNA methylation and transcription of key lineage-specific genes *in vivo* is preserved in primary culture

In order to tractably examine the impacts of age and microenvironment on lineage specificity, we developed LEP- and MEP-marker probe sets that enabled exploration of the relationships between promoter methylation and gene expression. These were used in functional cell-based experiments that require small numbers of cells and allow many replicates. To maintain consistency, all experiments used fourth passage (4p) pre-stasis finite lifespan HMEC from discarded reduction mammoplasty tissue for cell function studies [6]. The tissues were obtained from women, who do not have breast cancer, differing in chronological age at the time of surgery. The so called in vitro “replicative ages” are the same 4p at (Table S1).

**Figure 1:**
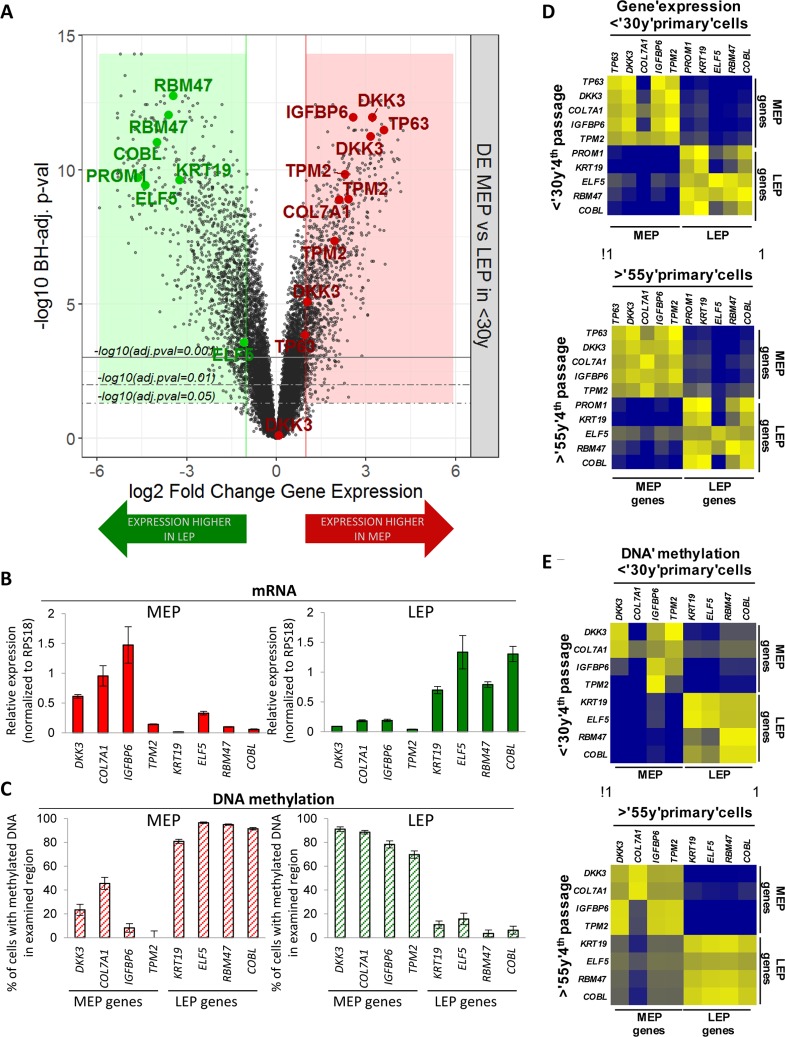
Lineage-specific gene expression and promoter methylation is consistent between HMEC in vivo and pre-stasis cultures (**A**) Volcano plot based on differential expression (DE) analysis of 24,965 Illumina gene probes (19,499 mapped genes) in 4p MEP and LEP from <30y subjects by beadchip expression array. Y-axis indicates –log10 Benjamini-Hochberg (BH)-adjusted p-values from significance analysis and x-axis shows log2 fold change (LFC) in gene expression. Colored regions and lines highlight fraction of genes which show lineage-specific differential expression (absolute log2 fold change ≥ 1 and BH adj. p-val < 0.05, < 0.01, < 0.001) with negative LFC values (green area) indicating higher expression in LEP and positive LFC values (red area) higher expression in MEP. LEP-specific (green circle) and MEP-specific (red circle) genes used as lineage-specific probesets are annotated (19 Illumina gene probes). Validation of lineage specific (**B**) gene expression in and (**C**) corresponding promoter DNA methylation in FACS enriched MEP and LEP, using qPCR-based lineage gene probe sets. Lineage specific expression was inversely correlated with DNA methylation status in the promoter. Differential expression and methylation in each gene were significant (p < 0.01). Expression data was normalized to expression of RPS18. Data were shown by mean ± SEM. Correlation of (**D**) Lineage-specific gene expression and (**E**) promoter DNA methylation status in MEP and LEP between 4p HMEC strains and uncultured cell dissociated from organoids. Pearson's correlation value of gene expression and DNA methylation between organoids and 4p HMEC were 0.9670 and 0.9333, respectively.

Transcriptome-wide differential expression analysis (Illumina HumanHT-12 v4 BeadChips Set 1, n=24,965 gene probes, m=19,499 mapped genes) of FACS-enriched CD10+/CD227- MEP and CD10-/CD227+ LEP from 4 different HMEC strains were used to identify lineage specific genes. Genes selected for use as probes showed >3-fold differential expression (DE) between LEP and MEP from women <30y (Benjamini-Hochberg, BH, adj. p-val < 0.05) (Fig. 1A) while also having CpG islands in their 5′ region within 5kb of the ATG start codon. These genes did not exhibit culture adaptive expression changes. Probe sets were designed to facilitate qPCR analyses of gene expression and promoter methylation of DNA by McrBC methylation sensitive enzyme digestion. DKK3, COL7A1, IGFBP6 and TMP2 were selected as MEP marker genes, and KRT19, ELF5, RBM47 and COBL were selected as LEP marker genes. These genes showed lineage-specific expression (Fig. 1B) that was inversely correlated with promoter DNA methylation (Fig. 1C) in LEP and MEP from eight different women <30y, suggesting that transcription of these genes was at least partly regulated by DNA methylation. The LEP and MEP markers PROM1 and TP63, respectively, also were used as well-known mammary epithelial lineage markers that do not appear to be controlled by methylation in CpG islands within 5kb of the start codons. The probe sets showed excellent correlation between 4p HMEC and FACS-enriched LEP and MEP from uncultured human mammary epithelial organoids, both in terms of lineage-specific gene expression (Fig. 1D, r^2^=0.96) and DNA methylation (Fig. 1E, r^2^=0.93). Results from the probe-based assays were comparable to the methylation and expression data from in the NIH Roadmap Epigenomics Project (Fig. S1) [19]. Thus these lineage-specific probe sets were uncultured mammary epithelia validated both with in vivo samples and in publicly available data.

### Myoepithelial cells maintain phenotypic fidelity of luminal cells

**Figure 2:**
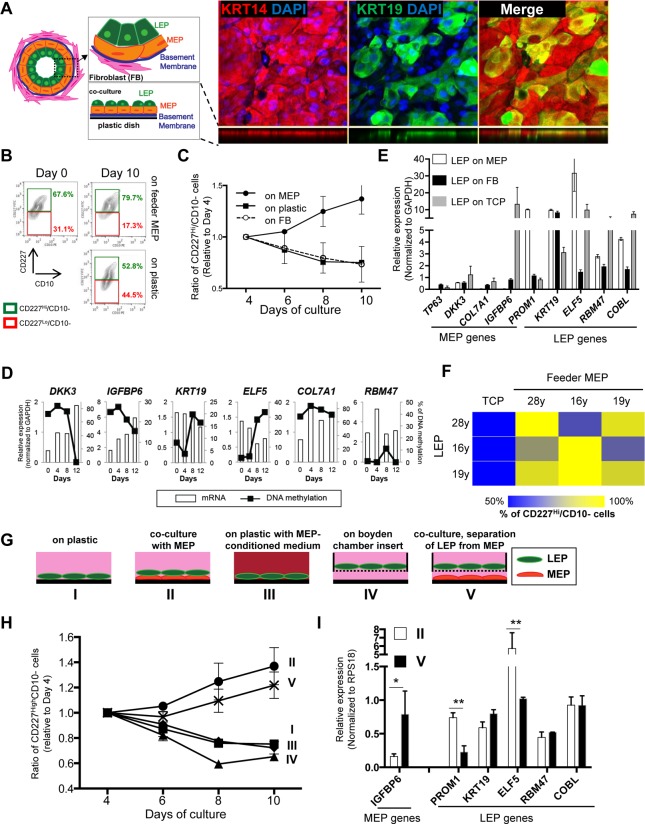
Apical surfaces of myoepithelial cells provide a robust microenvironment for maintenance of the luminal lineage (**A**) Schematic model of human mammary glands, co-culture and immunofluorescent staining in co-culture. Six days co-culture of LEP with MEP from 19y old were stained with antibodies against KRT14 (red) and KRT19 (green). Nuclei were stained with DAPI (blue). Orthogonal views were shown below each image. (**B**) Contour plots of CD10 and CD227 expression, as measured by FACS, comparing luminal cell populations on tissue culture plastic or co-cultured on a layer of MEP. LEP populations were maintained better after 10 days in co-culture (78.7%) compared to TCP (52.8%). (**C**) Line graphs showing the relative proportion of CD227+ LEP over time in co-culture with isogenic MEP or isogenic fibroblast (FB) feeders, or on plastic. (n=3) (**D**) Gene expression in LEP (white bars) and promoter methylation (black line) over 12 days of culture on TCP. LEP genes tend to be reduced with increasing methylation, whereas MEP gene expression is increased with reduced methylation. (**E**) Bar graphs showing differences in LEP- and MEP-specific gene expression in LEP cultured on FB feeders (black), TCP (gray) or on MEP feeders (white). Mean ±SD, normalized to expression of *GAPDH* (n=3). LEP gene expression is not maintained on FB feeders nor on plastic. (**F**) Heatmap showing the proportion of LEP, measured with FACS, remaining after 10 days co-culture with different combinations of MEP feeders from women <30y. (**G**) Schematic outlines that represent conditions I-V. (**H**) Line graphs showing proportion of LEP that is maintained over 10 days cultured in conditions (I-V). (n=3) (**I**) Bar graphs showing expression of lineage specific genes in LEP cultured 10 days in conditions II and V. Condition II was the only one to maintain a complete LEP phenotype over time. Mean ±SD, normalized to expression of *RPS18* (n=3). * and ** showed statistical significances at p<0.05 and p<0.01, respectively.

*In vivo*, LEP organize into an inner layer that borders a lumen, and is surrounded by MEP, which produce basement membrane components (Fig. 2A). To study LEP-MEP interactions, we established a biologically relevant LEP/MEP co-culture system that mimicked a LEP-microenvironment in mammary glands. LEP from <30y HMEC were cultured on top of completely confluent MEP feeder layers. When mammary epithelial cells are cultured they produce ECM that adsorb to the plastic and establish an apical (the side facing up) and a basal surface (side attached to ECM) [20]. We used this property of MEP to advantage and established a co-culture method that recapitulates a bilayered epithelia as shown in Figure 2A. Maintenance of CD227^+^/CD10^−^ LEP cellular phenotype was determined by FACS measurement of these surface protein markers over 10 days in culture (Fig. 2B). In LEP/MEP co-cultures, the proportion of LEP, defined as the fraction of cells maintaining CD227^+^ expression levels, increased steadily by day 10, showing a 15% increase compared to day 0, whereas the proportion of LEP declined steadily when cultured directly on tissue culture plastic (TCP) (Fig. 2B and 2C). The proportion of CD227^+^ LEP decreased at a rate similar to culture on TCP when co-cultured on isogenic breast stromal fibroblasts (Fig. 2C). Furthermore, LEP genes were down-regulated while *IGFBP6*, a MEP gene, increased in LEP on TCP and fibroblast co-cultures, whereas LEP genes remained high and MEP genes were suppressed in LEP grown on MEP feeders (Fig. 2E). Changes in expression of number of LEP- and MEP-specific genes in LEP that were cultured on TCP were anti-correlated with methylation changes (Fig. 2D). The other MEP genes also increased expression in LEP cultured in TCP and fibroblast microenvironments, but *IGFBP6* proved to be particularly responsive, and thus most useful in subsequent experiments. To determine whether the ability to maintain LEP was dependent on isogenicity, co-cultures were established with LEP and MEP from three individuals aged 16-28y in the nine possible combinations. In all cases, maintenance of LEP on MEP co-cultures was superior to LEP on plastic, and was independent of the individual (Fig. 2F).

To understand what type of physical interactions between LEP and MEP were required, LEP were cultured in a number of defined conditions. LEP from a 19y woman were cultured on (I) plastic, (II) co-cultured with isogenic MEP, (III) plastic with MEP-conditioned medium, (IV) mono-cultured with LEP attached to Boyden chamber inserts or (V) co-cultured with isogenic MEP in which LEP were physically separated by a Boyden chamber insert (Fig. 2G). CD227 and CD10 expression was monitored every two days by FACS (Fig. 2H). Co-cultures II and V, regardless of direct or no-direct contact of LEP with MEP, showed increased proportions of CD227+ cells over time, whereas LEP declined in mono-culture conditions (I, III and IV) (Fig 2H). Although the CD227+ cells population increased in both co-culture conditions II and V, the no-direct contact co-culture (V) showed reduced expression of *PROM1* and *ELF5* and increased *IGFBP6* expression compared to direct cell-cell contact (II)(Fig 2I), suggesting that only a partial LEP phenotype was maintained in co-cultures where MEP and LEP were physically separated (in this case by 900 μm). These experiments revealed that TCP does not provide an optimal micro-environment for maintaining the LEP phenotype even in presence of MEP-conditioned media. Indeed, LEP on TCP are eventually reprogrammed to more MEP-like states, and senesce more quickly than MEP (not shown), which serves as an extreme example of how micro-environment can negatively impact the LEP phenotype. These data show that direct LEP-MEP contact provided crucial microenvironment components for maintenance of the LEP phenotype. That stromal fibroblasts – a cell type that is ostensibly at least one cell-diameter removed from LEP in vivo, did not provide a satisfactory micro-environment for main-tenance of LEP underscores the specificity of the LEP-MEP interactions for maintaining the LEP phenotype.

### Loss of luminal-fidelity with age coincides with disruption of the relationship between promoter methylation and gene expression

**Figure 3:**
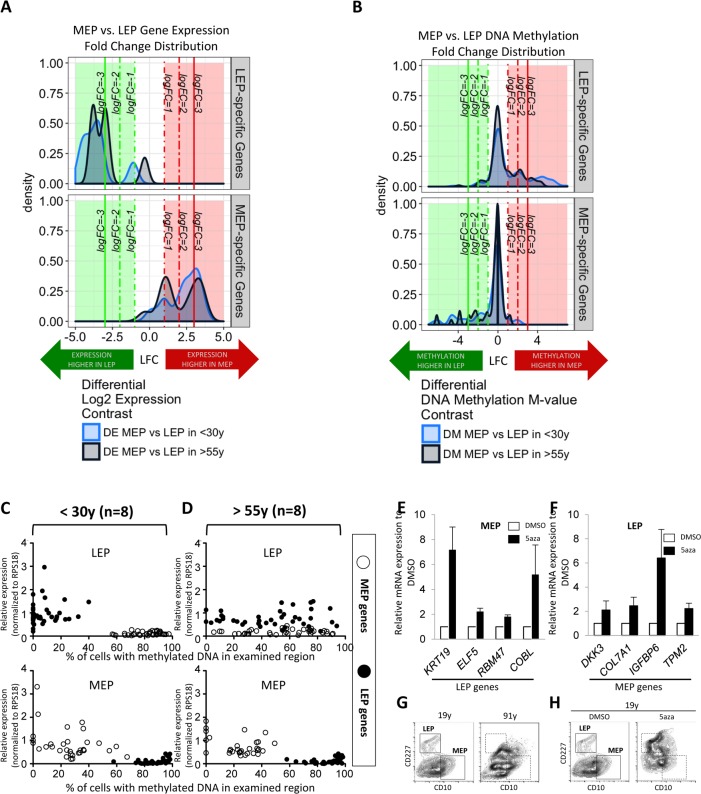
Age-dependent gene expression in luminal cells is associated with specific DNA methylation patterns LEP- and MEP-specific probe sets were used to identify age-dependent changes in lineage-specific gene expression and DNA methylation patterns in FACS enriched 4p LEP and MEP. Corresponding expression of probeset genes in LEP and MEP cells from 9 different 4p HMEC strains representing <30y and >55y age groups in Illumina HumanHT-12 v4 BeadChips (Set1) were assayed for lineage-specific differential expression (DE) between MEP and LEP across 19 Illumina gene probes (**A**). Infinium 450K methylation arrays were then used to evaluate lineage-specific differential methylation (DM) based on methylation M-values of probeset genes across 247 CpG sites for the same subjects (**B**). Kernel Density Estimates (KDE) of distributions of log2 fold changes (LFC) in expression (**A**) or DNA methylation (**B**) between MEP vs. LEP in <30y (light blue) and >55y (dark blue) subjects for LEP-specific (top panel) and MEP-specific genes (bottom panel) are shown. Colored regions and lines highlight fraction of genes or CpG sites which show lineage-specific differential expression or methylation respectively (≥ 1-, ≥ 2-, ≥ 3- absolute LFC and Benjamini-Hochberg, BH, adj. p-val < 0.05, < 0.01, < 0.001), with negative LFC values (green area) indicating higher expression/methylation in LEP and positive LFC values (red area) higher expression/methylation in MEP. (**C** and **D**) Dysregulation of lineage specific gene expression with age in LEP was associated with age-dependent DNA methylation patterns. The relationship between expression and methylation of lineage-specific genes in FACS enriched LEP and MEP from women (**C**) <30y or (**D**) >55y, is visualized using dot plots. LEP probes are shown as filled circles, MEP probes are shown as open circles. A change in the lineage-specific relationship was most prominent in older LEP. Eight strains were used for each age group, expression data were normalized to expression of *RPS18*. Bar graphs showing expression of (**E**) LEP genes in MEP treated with 5′aza, and (**F**) MEP genes in LEP treated with 5′aza, showing that these lineage specific genes were regulated in part by DNA methylation. (**G**) Contour plots representing CD10 and CD227 expression measured by FACS on HMEC from a 19y and a 91y woman, which are representative of the phenotypes typically observed in these extreme age groups. Corresponding areas were shown with dotted line boxes. (**H**) CD10 and CD227 expression in HMEC from a 19y woman treated with DMSO or DMSO+5′aza at 15 μM for 48h. Young HMEC phenocopied older HMEC following 5aza treatment. Gates used to delineate lineages are indicated with boxes.

To determine the consequences of age on fidelity of lineage-specific gene expression, we interrogated the corresponding expression (Illumina HumanHT-12 v4 BeadChip Set 1) and DNA methylation (Infinium Human Methylation 450K BeadChip) patterns of the selected lineage-specific probeset genes in LEP and MEP cells from 9 different HMEC strains representing <30y and >55y age groups. Differential expression (DE) analysis focused on our probeset genes (n=19 Illumina probes) shows that there is a shift towards smaller fold-differences between LEP and MEP genes in the >55y group compared to the <30y group (Fig. 3A). That is, fidelity with which lineage-specific genes are expressed decreased with age, LEP and MEP looked more alike.

Differential methylation (DM) analysis of M-values (the log2 ratio of methylated to unmethylated probes) at CpG sites associated with the probeset genes (n=247 Illumina CpG sites) revealed a gross inverse relationship bet-ween expression and DNA methylation; LEP-specific genes had higher methylation levels at these loci in MEP, and vice versa (Fig. 3B). The distribution of fold changes in methylation decreases in magnitude of lineage-specific differences in the >55y group compared to the <30y group (Fig. 3B). The effect of age is most evident in the LEP-specific probeset genes. Viewing the methylation architecture of these probeset genes at the resolution of annotated gene regions, we find age-dependent changes in methylation mainly occur in the genes’ regulatory regions, e.g. transcription start sites (TSS), 5′-untranslated region (UTR), and some in the 1^st^ exon (Fig. S2A).

Consistent with a loss of lineage fidelity, but not specificity, qPCR results showed *KRT19* and *ELF5* significantly decreased expression (Fig. S2B), while MEP-specific genes modestly increased in LEP from the >55y women (Fig. S2C). Age-dependent gene expression patterns were similarly associated with inverse changes in promoter DNA methylation when evaluating McrBC digested gDNA with qPCR (Fig. S2B and S2C). The gene expression and DNA methylation patterns in MEP were stable during aging, with the exception of *RBM47* and *DKK3*, which show modest increased and decreased in expression with age, respectively. When the expression/methylation relationships of all the lineage-specific genes were viewed together using the qPCR probeset, the relationship between age, lineage, and lineage-specific gene regulation became most apparent. Both younger MEP and LEP, as well as older MEP, show tightly regulated lineage-specific gene expression that were inversely correlated with DNA methylation (Fig. 3C and 3D). However, in older LEP, the LEP-specific pattern of gene expression and methylation were significantly disrupted (Fig. 3D).

We next asked whether artificial disruption of the lineage-specific gene expression/methylation patterns in normal HMEC could recapitulate the biochemical loss of lineage fidelity in phenotypes that arise with age. After HMEC from a 19y woman were treated for 48h with 5-aza-2′dC (5aza), the inhibitor of DNA methyltranferases, the expression of otherwise suppres-sed lineage-specific genes were observed in both MEP (Fig. 3E) and LEP (Fig 3F). HMEC from women <30y typically exhibit distinctive CD10+/CD227- MEP and CD10-/CD227+ LEP populations by FACS analysis, and the divisions between the two populations are typically become less obvious in HMEC from older women (representative examples of <30y and >55y 4p HMEC in Fig. 3G). Treatment of the 19y HMEC with 5aza phenocopied a lineage distribution more characteristic of HMEC from a much older person (Fig. 3H). Although the effects of using 5aza are non-specific, the approach showed that disruption of the DNA methylation-gene expression relationship during aging may partly explain loss of lineage fidelity. Taken together we speculate that changes in methylation of LEP-specific genes is fundamental to loss of fidelity with age in this lineage.

### Age-related lineage fidelity in luminal cells is a switchable state that is determined by the age of myoepithelial cells

**Figure 4:**
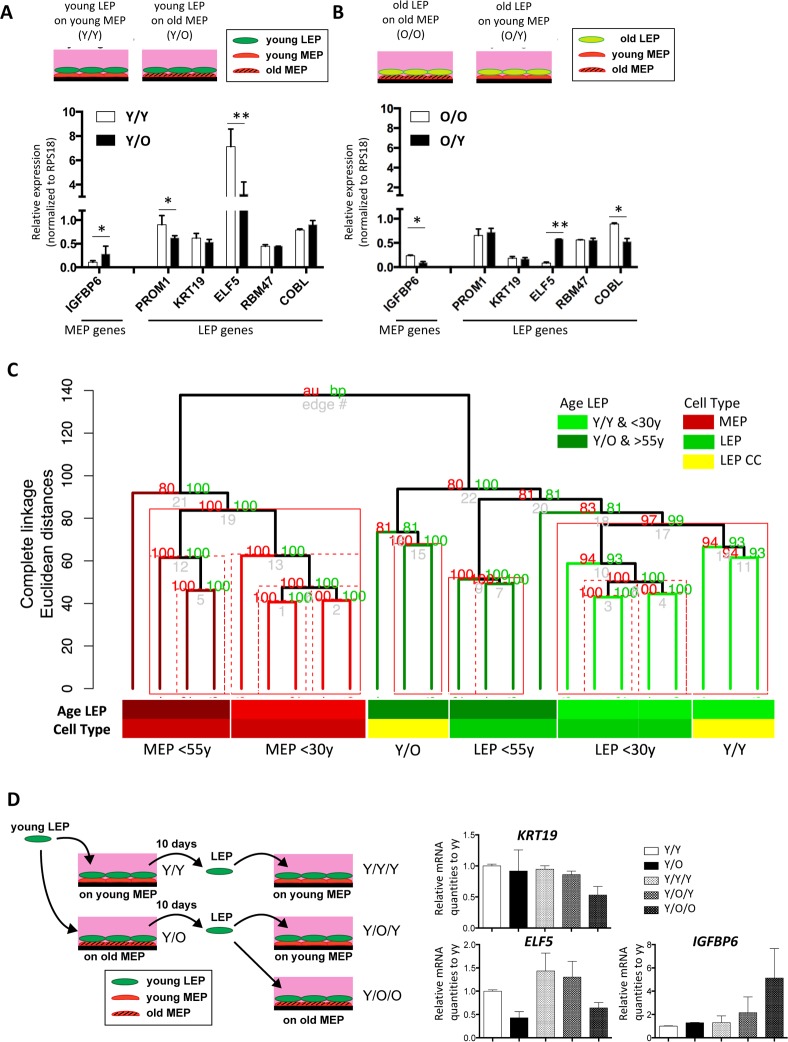
Chronological age of the apical microenvironment determines age-dependent gene expression patterns in luminal cells (**A**) Schematic shows co-culture conditions VI and VII, with young LEP atop of young or old MEP, respectively. Bar graph shows differences in LEP-specific gene expression, and IGFBP6 a MEP-specific gene, in young LEP after 10 days culture on young (white) or old (black) MEP feeders. Mean ±SD, normalized to expression of RPS18. (**B**) Schematic shows co-culture conditions, with old LEP atop of old or young MEP, respectively. Bar graph shows differences in LEP-specific gene expression, and in *IGFBP6* a MEP-specific gene, in old LEP after 10 days culture on young (white) or old (black) MEP feeders. Mean ±SD, normalized to expression of *RPS18* (n=3). * and ** showed statistical significances at p<0.05 and p<0.01, respectively. (**C**) Unsupervised hierarchical clustering of <30y LEP in Y/Y (n=3) and Y/O (n=3) co-cultures in parallel with <30y (n=5) and >55y (n=4) 4p LEP and MEP isogenic to the MEP strains used in co-culture (Illumina HumanHT-12 v4 BeadChips Set2). Clustering was performed on transcriptome-wide log2 gene expression levels (n=26,599 gene probes, m=20,577 mapped genes) using Euclidean distance measures and complete linkage. Percent Approximately Unbiased (AU) p-values in red, and percent Bootstrap Probability (BP) in green are calculated and annotated above each cluster (pvclust R package). Clusters with AU > 95% are highlighted by red rectangles, solid red rectangles denotes largest cluster supported by data. (**D**) Schematic of experimental outline for extended co-cultures. LEP were separated by FACS after 10 days co-culture either with young MEP (Y/Y) or old MEP (Y/O). LEP from Y/Y and Y/O were further co-cultured with young MEP (Y/Y/Y and Y/O/Y) or older MEP (Y/O/O). (**E**) Bar graphs showing gene expression levels of KRT19, ELF5 and IGFBP6 in LEP following the 7-day culture experiments. Expression was normalized to expression of RPS18 and shown by relative expression to those of Y/Y.

**Figure 5:**
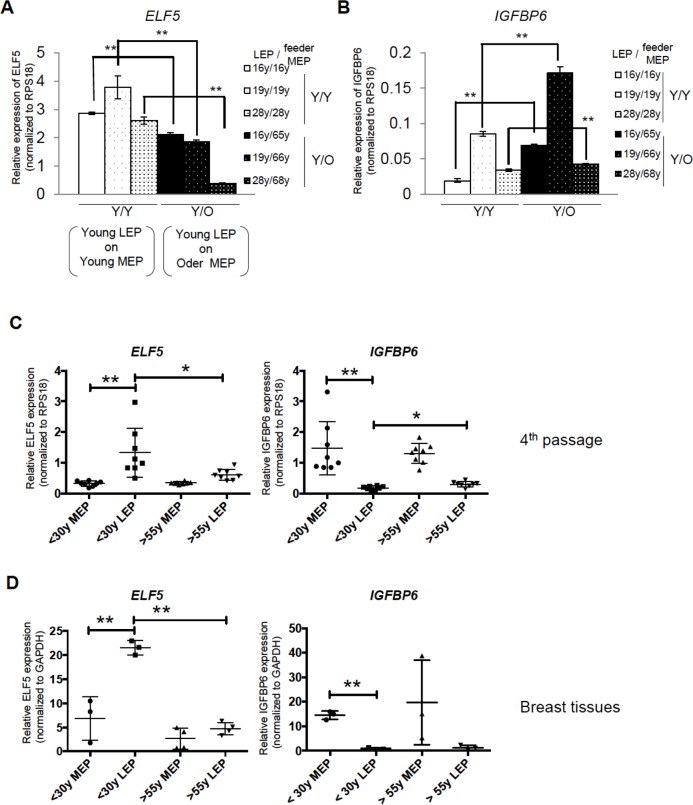
In vivo and primary cell age-dependent *ELF5* and *IGFBP6* expression were recapitulated in mixed age co-culture Mixed combinations of MEP and LEP from different aged donors demonstrates that age is the important determinant of *ELF5* and *IGFBP6* expression. Bar graphs showing (**A**) *ELF5* and (**B**) *IGFBP6* gene expression after 10 days in co- culture with different combinations of young LEP on young or old feeder MEPs. Gene expression was normalized to *RPS18*. (**C** and **D**) Age-dependent expression levels of the two genes in co-culture recapitulate 4p and primary HMEC. Lineage-and age-dependent *ELF5* or *IGFBP6* gene expression was shown by dot plots in (**C**) 4p HMEC and in (**D**) breast tissues. * and ** showed statistical significances at p<0.05 and p<0.01, respectively.

Because contact with MEP is important for the maintenance of LEP lineage specificity, we hypothesized that MEP from women of different age groups could impose age-dependent gene expression patterns in LEP. LEP from women <30y were co-cultured on isogenic MEP ((Y)oung/Y) or MEP from women >55y (Y/(O)lder). LEP- and MEP-marker gene expression was measured in LEP after 10 days of co-culture. LEP cultured on MEP from younger women (Y/Y) maintained robust LEP gene expression and suppressed MEP genes. However, in LEP cultured on MEP from older women (Y/O), expression of *PROM1* and *ELF5* decreased and *IGFBP6* increased (Fig 4A). In non-isogenic co-culture experiments, LEP from 3 young HMEC strains, cultured on MEP from 3 younger or 3 older women consistently showed that the age of MEP determined *ELF5* and *IGFBP6* levels in LEP (Fig. 5A and 5B).

We next determined whether LEP from older women could be rejuvenated in a younger microenvironment. On isogenic MEP (O/O), older LEP exhibited high *IGFBP6* expression and low levels of *ELF5* consistent with older primary LEP (Fig. 4B). In contrast, when cultured on younger MEP (O/Y), *ELF5* expression was increased 7-fold and *IGFBP6* decreased to background levels compared to LEP in O/O co-culture (Fig. 4B). The age-dependent MEP-imposed gene expression in LEP was consistent with age-dependent expression in FACS-enriched LEP from both 4p HMEC strains (Fig. 5C) and directly from breast tissue (Fig. 5D).

We next examined transciptome-wide expression of <30y LEP cells in either Y/Y (n=3) or Y/O (n=3) co-cultures in parallel with 4p LEP and MEP from both <30y (n=5) and >55y (n=4) strains isogenic to the MEP strains used in co-culture (Illumina HumanHT-12 v4 BeadChips Set 2). Unsupervised hierarchical clustering based on transciptome-wide gene expression levels (n=26,599 gene probes, m=20,577 mapped genes) show that the co-cultured LEP expression profile is deter-mined by the age-group of the MEP used for co-culture. Clustering of <30y LEP in Y/Y co-culture with <30y 4p LEP is highly supported (Approximately Unbiased, AU, p-val > 0.95), and separate from >55y 4p LEP and the corresponding isogenic <30y LEP in Y/O co-culture (Figure 4C).

Because down-regulation of *KRT19* in LEP with age is observed by immunostaining in human breast tissue and in 4p cultured cells, and is one of the most prominent age-related phenotypes that we have identified so far [6], it was unexpected that reduction of *KRT19* mRNA was not detected in mixed-age co-culture (Fig. 4A). We hypothesized that longer time scales might be necessary to observe broader phenotypic changes. Consequently, we examined the effect of protracted exposure to the aged microenvironments on the expression of *KRT19*, *ELF5* and *IGFBP6* in LEP (Fig. 4D). Young LEP were re-isolated after 10-days co-culture with MEP, then cultured on fresh (Y or O) MEP feeders for a further 7 days. Under prolonged Y/O/O exposures, *ELF5* and eventually *KRT19* were down-regulated, and *IGFBP6* was up-regulated (Fig. 4E). The pattern of *ELF5* and *IGFBP6* expression in the Y/O/Y conditions followed the age of the MEP donor and *KRT19* remained high (Fig. 4E). Thus, at least a subset of age-dependent gene expression patterns in LEP are malleable, able to be directed by the age of the MEP in the microenviron-ment. The extent of this age-dependent reprogramming of the LEP, i.e. the number of lineage-specific genes and their degree change in expression, was further dependent on the duration of exposure to micro-environments.

### Microenvironments impose age-specific promoter DNA methylation states at *ELF5* and *IGFBP6* loci

**Figure 6:**
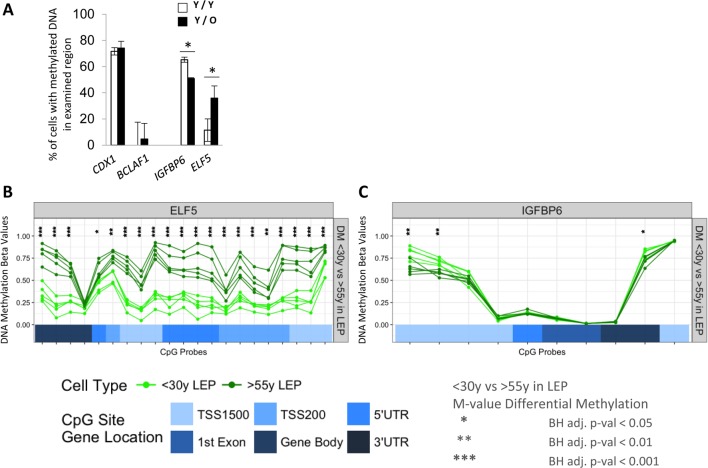
Age of the apical microenvironment is a determinant of *ELF5* and *IGFBP6* promoter DNA methylation states (**A**) Bar graphs showing the percent of *IGFBP6* and *ELF5* methylated promoter DNA in LEP after 10 days of culture on young (white) or old (black) MEP feeders. *CDX*1 and *BCLAF1* are hyper- and hypomethylated gene controls. Data are presented as mean ±SD (n=3). * indicates statistical significances at p<0.05. DNA methylation analyses of (**B**) *ELF5* and (**C**) in *IGFBP6* using Infinium 450K methylation arrays. Analysis assessed percentage methylation (beta-values) and age-specific differential methylation (DM) across CpG sites in these genes for <30y LEP (green) and >55y LEP (dark green). DNA methylation beta-values across CpG sites are plotted in order of their chromosomal mapping, and range from 0-1 denoting hypo- (β-val < 0.25), hemi- (0.25 < β-val < 0.75) and hyper-methylated (β-val > 0.75) methylation levels. Corresponding annotated locations of CpG sites respective to gene regions: TSS1500, TSS200, 5′UTR, 1^st^ Exon, Gene Body and 3′UTR (shades of blue) are shown in tracks below. Significance of age-specific differential methylation based on corresponding M-values between <30y and >55y LEP are denoted by asterisks: Benjamini-Hochberg, BH-, adj. p-val (*) < 0.05, (**) < 0.01, (***) < 0.001.

We next determined if exposure of LEP to the different aged MEP was associated with also changes in DNA methylation in the promoter regions. *ELF5* and *IGFBP6* were the focus of these experiments because age-dependent changes in expression were reliably linked to changes in promoter methylation. DNA methylation was examined in young LEP co-cultured with young (Y/Y) or old (Y/O) MEP for 10 days. *CDX1* and *BCLAF1* were used as hyper- and hypo-methylation control genes, respectively, which did not change in any of our co-culture conditions (Fig. 6A). DNA methylation in LEP in the regulatory regions of *ELF5* increased 30±9% in Y/O cultures and methylation at the *IGFBP6* promoter decreased 13±0.7% compared to Y/Y (Fig. 6A). These changes in methylation measured with McrBC digests in co-culture were consistent with age-dependent changes in *ELF5*- and *IGFBP6*- DNA methylation at the gene regulatory regions (Infinium Human Methylation 450K BeadChip) of <30y vs. >55y FACS enriched 4p LEP (Figure 6B-6C). In 4p LEP, differences in corresponding methylation beta-values, which measure percentage of methylation, show large age-specific increase in methylation levels in *ELF5* across numerous CpG sites in the gene regulatory region from hypo-methylated (β-val < 0.25) in <30y LEP to hemi- (0.25 < β-val < 0.75) and hyper-methylated (β-val > 0.75) in >55y LEP. Furthermore, these changes in ELF5 methylation levels in >55y LEP shift towards more MEP-like methylation levels (compare Fig. 6B, Fig. S3A), showing loss of lineage-specific methylation with age. A smaller, though significant, decrease in methylation levels in *IGFPB6* regulatory region is observed with hyper-methylated sites in <30y LEP becoming hemi-methylated in >55y LEP (compare Fig. 6C, Fig. S3B). These differentially methylated regulatory regions in *ELF5* and *IGFBP6* correspond to either annotated enhancer element regions or DNaseI hypersensitivity sites, suggesting that tran-sition between hypo-, hemi- and hyper-metylation states in these regions could have a significant effect on transcription levels (Fig. S3A and S3B). Differential methylation between <30y and >55y 4p LEP for the remaining probeset genes are shown in Fig. S4A-S4F.

**Figure 7:**
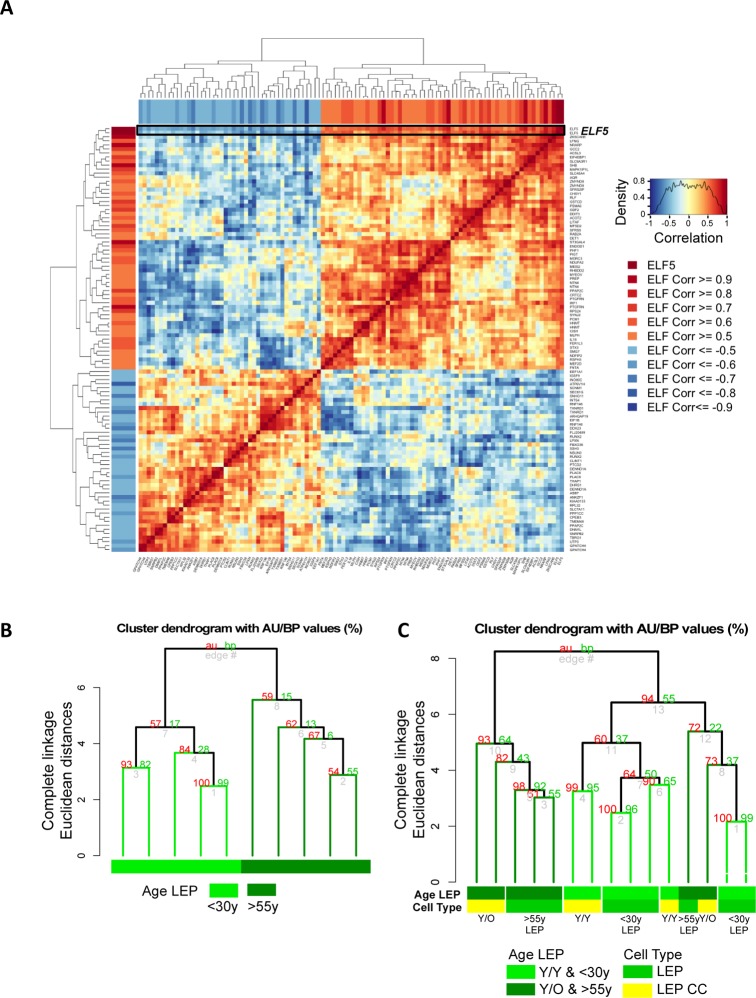
Microenvironment-imposed reduction of *ELF5* causes an entire network of genes to change Age‐related changes in *ELF5* are associated with age‐specific changes in *ELF5*‐target genes in the LEP lineage. (**A**) Gene‐gene correlation matrix of ELF5 (2 gene probes) and 92 *ELF5*‐target genes (103 gene probes) found to have absolute correlation ≥ 0.5 with *ELF5* in LEP from <30y and >55y age groups across 9 HMEC strains. Annotated in both the row and column bars of the correlation matrix is each *ELF5*‐target gene probe's correlation value to the ELF5 probes. (**B**) Hierarchical clustering based on log2 expression levels of *ELF5* and the anti‐/correlated *ELF5*‐target genes in <30y and >55y 4p pre-stasis LEP, (**C**) and in Y/Y (n=3) and Y/O (n=3) co‐cultures with <30y (n=5) and >55y (n=4) 4p LEP isogenic to the MEP strains used in co‐culture, using Euclidean distance measures and complete linkage. Percent Approximately Unbiased (AU) p-values denoted in red, and percent Bootstrap Probability (BP) in green are calculated and annotated above each cluster.

Methylation of the promoters should facilitate stabilization of microenvironment-imposed gene expression. Furthermore, when the regulated gene product is a lineage-specific transcription factor, we should be able to measure changes in downstream gene networks. As a transcription factor, *ELF5* warranted further examination and we asked whether age-related decline of *ELF5* alters expression of target genes. *ELF5* target genes were previously identified by ELF5-ChIP in the breast cell line T47D (499 probes, 429 unique genes at FDR < 0.05, t = 48HR) [21]. Of this ELF5-ChIP gene set, 323 of the prospective *ELF5*-target genes were in our Illumina HumanHT-12 v4 BeadChips (Set 1) corresponding to 528 gene probes. Gene-gene correlation analysis showed 92 target genes corresponding to 105 gene probes to be correlated or anti-correlated with *ELF5* expression in 4p LEP from 9 different HMEC strains from both <30y and >55y age groups (absolute correlation ≥ 0.5, Fig. 7A). Pre-stasis 4p LEP clustered according to age based on *ELF5* and *ELF5*-target gene expression (Fig. 7B). Furthermore, young LEP co-cultured with young MEP (Y/Y) clustered with <30y LEP, while two out of three young LEP co-cultured with old MEP (Y/O) clustered with a majority of >55y LEP (Fig. 7C). This suggests that age-related changes in *ELF5* expression impact known target genes downstream of *ELF5* in LEP in an age-dependent manner, and that these changes can be driven by cues from the MEP microenvironment. Taken together, these data converge on the conclusion that the age of MEP-generated microenvironments is a determinant of promoter DNA methylation states, which may drive, and stabilize, some of the age-related gene expression phenotypes in LEP.

## DISCUSSION

Here we show that the apical surface of human mammary myoepithelial cells (MEP) comprise a microenvironment that maintains luminal cell (LEP) fidelity. Intrinsic changes that occur in MEP during aging can exert aging phenotypes on neighboring LEP by altering gene expression networks, revealing the possibility that aging LEP phenotypes occur through a cell non-autonomous mechanism. LEP may consequent-ly be more sensitive to microenvironment changes than MEP, exemplified by the observation that they are easily lost during culture on TCP and, as we show here, reprogrammed to a more MEP-like phenotype. Fibro-blasts are commonly used to establish co-cultures, because they are thought to provide some minimal microenvironment components, but we show here, even isogenic normal fibroblasts performed no better than TCP at maintaining LEP. MEP already were known to be contractile, tumor suppressive, and crucial for maintaining polarity of the epithelia [22]; that the LEP phenotype was maintained in culture on MEP-feeder layers revealed a novel role of MEP in maintaining the fidelity of a different lineage of epithelial cell. This new found role opens an interesting possibility to explore the role of MEP in aging phenotypes that we observed in LEP, which we previously characterized as the acquisition of traits otherwise only seen in MEP. Indeed, mixed-age LEP-MEP co-cultures showed that age-dependent gene expression and methylation states in LEP were malleable, driven between young and old phenotypes in accordance with the chronological age of the MEP. Taken together, we provide evidence that the luminal epithelial phenotype is exquisitely sensitive to microenvironment conditions, and that states of aging are cell non-autonomously communicated through microenvironment cues over distances of at least one cell diameter.

We focused on the communication between the LEP and MEP lineages because most breast cancers are thought to originate from cells that reside in the luminal or supra-basal compartments. Previously, it was difficult to maintain the LEP lineage in culture. A breakthrough crucial to revealing that the chronological age of MEP influenced epigenetic patterns in LEP was our discovery that direct contact with the apical surface of MEP, but not isogenic stromal fibroblasts, was sufficient to maintain LEP for multiple passages. These experiments have led us to hypothesize that age-related systemic microenvironment changes alter the cells that comprise breast tissue, which reciprocally alter their microenvironment, and ultimately trickle through the entire tissue altering the epithelial cells [23]. The tissue neighborhood where LEP reside represents the terminal-most node for information that may be transmitted from as far away as the circulation or stroma. Hormone changes exert systemic effects via endocrine mecha-nisms. Adipocytes and fibroblasts in breast stroma alter each other and the MEP, and the MEP alter the LEP and suprabasal progenitor cells via reciprocal juxtacrine and paracrine interactions. We hypothesize that these interactions change as breast tissue ages, leading to age-specific epigenetic and gene expression patterns. Based on our probe set, we did not observe MEP to show age-dependent mRNA expression changes like those in LEP. However, it is clear LEP lineage-fidelity is affected by age-dependent MEP microenvironments. We have previously reported that YAP cellular localization was altered by age [5], and it is possible that functional differences of MEP may be due to proteins that have age-dependent post translational modifications or localizations. Protracted exposures to the gradually changing microenvironments may turn temporary epigenetic changes into more permanent metastable ones by modification of DNA and histones. Ultimately these changes lead to loss of lineage-fidelity and decreased tissue function.

There have been limited model systems that allow examination of aging processes in human tissue contexts. The HMEC Aging Resource provides a growing collection of uncultured and cultured HMEC strains from women who ranged in age from 16 to 91 years, established from normal reduction mammoplasty tissue, for the purpose of examining consequences of aging in breast [6]. We found age- and lineage-depen-dent DNA methylation and gene expression patterns to be consistent between primary uncultured and early passage HMEC, and with publicly available gene expression data, providing multiple sources of validation for our cellular system. Evidence that there is a relationship between microenvironment and specific epigenetic states has been tenuous due to a paucity of examples. These studies have shown that, e.g. cells placed in embryonic microenvironments [24] or in tumor core or peripheral regions [25] have specific patterns of DNA and histone modifications, and histone and DNA methylation states in carcinoma cell lines can be modulated by 2-D versus 3-D culture conditions [26, 27]. Oyer et al showed that prolonged repression of a gene promoter by an inducible negative regulator resulted in more DNA methylation at that locus, leading to maintenance of the repressed state [28]; multiple epigenetic repressors often are co-located [29, 30]. Importantly, de novo gene methylation is not required for silencing, and it was hypothesized to be a secondary event that sustains modification made by other more rapid-acting forms of epigenetic regulation [31]. This notion is consistent with our findings. We previously reported that reduction in K19 in LEP is a hallmark of aging in breast. However, in culture we observed that whereas a number of LEP and MEP genes were altered rapidly within 10 days, reduction of K19 imposed by older MEP required at least 20 days. Changes in expression of *ELF5* and *IGFBP6* even were accompanied by changes in promoter methylation. Thus, protracted exposure to microenvironments gradually altered by aging may impose metastable transcription states by altering epigenetic regulators. Such a mechanism helps to explain the durability of age-related changes in cell function that lead to reduced tissue function.

The ability to modulate the effective age of LEP by changing the chronological age of their micro-environment required cell-cell contact, and the degree of modulation was dependent on the exposure-time length. In model organisms, aging phenotypes have been reversible by altering the circulatory and local tissue microenvironments in muscle [32], liver [32], heart [33], and CNS [34]. We have shown one means by which it is possible to rejuvenate cells of a human tissue using primary cell culture together with tissue recombination techniques. While this method will not work to understand how all systems in a body age, nor how the various tissues interact over long distances, it does model well the human mammary epithelium. Even if we did not understand all the signals coming from a distance, learning to control the LEP-MEP interaction could lead to a means of preventing age-related loss of lineage fidelity, which we hypothesize is central to age-related breast cancer susceptibility. LEP exist in vivo in a mainly, if not entirely, cell-cell contact-type micro-environment. Engagement with ECM is mainly a role for the MEP. As our in vitro cell system evolves, inclusion of stromal cell types, endothelial cells, and blood cells that are necessary to mimic chronic inflammation, will lead to an improved understanding of cell non-autonomous mechanisms of aging in human tissues.

Down regulation of *ELF5* with age also was reported in mammary glands of non-human primates [35], suggesting that it is a conserved consequence of aging. *ELF5* has crucial roles in embryogenesis, mammary development [36] and differentiation into ERα negative luminal lineages in mouse mammary glands [37]. Reduction of *ELF5* in mouse mammary epithelial pro-genitors leads to their accumulation, consistent with the relationship between *ELF5* down regulation with age and the accumulation of KRT14/KRT19 double positive mammary progenitors that we reported previously [6]. *ELF5* represses EMT by suppressing SNAI2 expression [38] and down regulating ESR1 [21], and down regulation of *ELF5* is detected in all stages of cancer progression including atypical ductal hyperplasia, ductal carcinoma in situ and invasive ductal carcinoma [39]. We did evaluate the possibility of age-associated EMT-like states in LEP, but we did not find convincing evidence of full-blown EMT. Only a few of the classical EMT-related genes followed the expected pattern (up: SNAI2, TWIST2, AHNAK; down: K19, MST1R), but many other genes that one might expect to change, such as vinculin and AXL, do not change. Perhaps one should not expect a full blown EMT in this context, as aging is not a disease state. Increased *ESR1* expression in LEP driven by decline of *ELF5* with age could be a possible mechanism to explain the increased ER expression in the normal breast with age [4], and may be relevant to the observation that the most common breast cancer subtypes in older women are ER+ luminal subtypes [40].

Epigenetic states associated with biological age may underlie the bias in cancer subtype. A majority of age-related breast cancers are luminal subtypes, and transcriptomes of luminal-type breast tumors from younger and older patients show age-dependent patterns that are not attributable to genomic variation [41]. We previously reported that immortalized HMEC from post-menopausal women were biased towards luminal subtypes, implicating age-related epigenetic state of the normal cells as a principal determinant of subtype [13]. It is tempting to speculate that once plasma estrogen and other hormones are greatly reduced after meno-pause, the mammary epithelia may find a means to repress a negative regulator of ERα, thus making the tightly controlled estrogen circuitry leakier. Estrogen signaling is a significant risk factor in Luminal A-type breast cancers, which represent 80% of age-related breast cancers [42]. We suggest that one consequence of *ELF5* down regulation with age is to dysregulate ERα expression in post-menopausal epithelia.

We provide evidence here for a microenvironment-based mechanism by which epigenetic states can be perpetuated throughout an epithelium via cell-cell interactions. Our experiments took advantage of differentiated LEP, which normally occupy the apical surfaces of MEP in vivo, to show the proof of concept. However, in vivo the multipotent and lineage-biased progenitors also occupy the region surrounded by MEP [43, 44] and so the mechanism is likely relevant to those cells as well. This is important because the cells-of-origin for breast cancer are thought to be the lineage-biased and multipotent mammary epithelial progenitors, and changes in their function may negatively impact the ability of the tissue to resist malignant transformation.

## METHODS

### Cell culture

Primary HMEC strains were generated and maintained as described previously [45, 46]. HMECs were grown in M87A medium containing cholera toxin and oxytocin at 0.5 ng/ml and 0.1nM, respectively. HMEC strains used in this study were listed in Table S1. In co-culture study, FACS-enriched MEP from 4^th^ passage HMEC strains or fibroblast cells were re-plated to 6-well plates and cultured until the cells were confluent. The cells were treated with mitomycin C (Santa Cruz Biotechnology) at 10μg/ml for 2.5h. Then, FACS-enriched LEP were seeded directly on the mitomycin C-treated cells or on a cell insert (BD) for separation from MEP cells. LEP in co-culutre were separated by FACS after 7-10 days for gene expression and DNA methylation analyses. 5-aza-2′-deoxycytidine (5aza) was added at 15 μM in culture medium. Cells were cultured for 48hr with culture medium containing 5aza and harvested for further analysis.

### Flow cytometry

Cells dissociated from organoids or primary HMEC strains were stained with anti-human CD227-FITC (BD Biosciences, clone HMPV) and anti-human CD10-PE (BioLegnend, clone HI10a) by following standard flow cytometry protocol. Co-cultured LEP was stained with anti-human CD227-FITC and anti-human CD10-PE and separated by FACS. Cells were sorted by FACSVantage SE or analyzed by FACSCalibur (BD Biosciences). Data were analyzed with FlowJo software.

### Quantitative gene expression analysis

Total RNA was isolated from FACS-enriched cells using Quick-RNA MicroPrep (ZYMO Research). cDNA synthesis was performed by iScript (BioRad) according to manufacturer's manual. Gene expression was measured by LightCycler 480 (Roche) with iTaq universal SYBR Green supermix (BioRad). Data were normalized to RPS18 or GAPDH by relative standard curve method or normalized by Vandesompele method [47]. ANOVA was used to test for statistical sig-nificance. Primers are listed in Table S2.

### DNA methylation analysis

Genomic DNA was purified using Quick-gDNA MicroPrep (ZYMO Research). Genomic DNA was digested with McrBC (New England BioLabs) and EcoRI (New England BioLabs), or *Eco*RI only as a control. DNA methylation was measured by real-time PCR using LightCycler 480. Amount of DNA methylation was normalized by internal primer control that targeted the DNA not containing CG dinucleo-tide. DNA methylation by McrBC method shows % of cells with methylated DNA.

### Statistical analysis

We examined the gene expression correlation between 4^th^ passaged HMECs and uncultured breast tissue cells using Pearson correlation. Figures 1D and E show the correlation of each gene's expression by colors. Yellow indicates that the trend of one gene expression across other genes is correlated to both HMECs and uncultured cells. Blue color indicates there are no correlations.

### Gene expression and DNA methylation analysis using Illumina beadchip

Total RNAs and genomic DNAs from LEP and MEP were isolated using Quick-RNA MicroPrep and Quick-DNA MicroPrep (ZYMO Rseach), respectively, after enrichment by FACS. Sample preparations for Illumina HumanHT-12 v4 Expression BeadChip array and HumanMethylation450 BeadChip array were performed in UCLA Neuroscience Genomics Core (UNGC).

Gene expression and DNA methylation of ten LEP and ten MEP samples from nine individuals (four young subjects <30 years old, five older subjects > 55 years old) were analyzed across two Illumina HumanHT-12 v4 Expression (Set 1) and two Infinium 450K Methylation BeadChip arrays. BeadChips were designed to have balanced, well-stratified distribution of samples for factors of interest (cell type and subject age) with one reference individual (240L MEP and LEP samples, <30 years old) shared between the two chips. Additionally, gene expression from six LEP co-culture samples (three biological replicates of 240L <30y LEP co-cultured on 240L <30y MEP, and three biological replicates 240L <30y LEP co-cultured on 122L >55y MEP) along with 18 samples of 4p LEP and MEP isogenic to the co-culture MEPs (five biological replicates of 240L <30y LEP and MEP, and four biological replicates 122L >55y LEP and MEP) distributed evenly across five Illumina HumanHT-12 v4 Expression (Set 2) were also analyzed.

Raw gene expression data from Illumina HumanHT-12 v4 BeadChips were pre-processed with Bioconductor limma package neqc function which performs normexpr background correction using negative control probes, log2 transformation and quantile normalization between arrays [48, 49]. Normalized data set were pre-filtered to remove gene probes with values less than negative control probes. This was done by calculating detection p-values using limma package detectionPValues function [48, 49], and only gene probes with detection p-values < 0.05 for at least 3 samples were retained. Potential batch effects between chips were checked using Principal Component Analysis (PCA).

DNA methylation measured using Infinium 450K Methylation BeadChips were analyzed using a custom R script. The arrays were read and normalized using the lumi package which return methylation M-values, the calculated log2 ratio of the intensities of methylated probe versus unmethylated probe [50]. Color bias adjustment was performed using lumiMethylC and simple scaling normalization (SSN) was performed using lumiMethylN functions [51]. Methylation beta-values, which is an approximation of the percentage of methylation of a given CpG site, and is calculated as the ratio of the methylated probe intensity and the overall intensity (sum of methylated and unmethylated probe intensities), were computed from M-values using m2beta function [50].

Differential expression (DE) analysis was performed on the pre-filtered normalized data set as a one-step method for handling batch effects is preferred for DE analysis. DE analysis was performed using Biocon-ductor limma package which applies an empirical Bayes approach proposed to provide more stable inference when number of arrays is small [49, 52]. Differential methylation analysis was performed on methylation M-values as M-values are found to be more statistically valid for differential analysis [50]. For both DE and DM analysis, a model was fitted with a coefficient for each of the four factor combinations (MEP/LEP cell type vs. young/old subject age group). Batch was added to the design matrix along with the factor combinations. Sample replicates, as well as paired nature of MEP/LEP samples were accounted for by blocking for individual in the duplicateCorrelation function [53]. Array weights were also computed and included in the model. The four contrast terms and an interaction term were considered for analysis. Empirical Bayes moderation of computed statistics was applied. Benjamini-Hochberg (BH)-adjusted p-values (Benjamini and Hochberg's method to control for false discovery rate) and log2 fold change statistics were calculated for each probe for both the lineage-specific (MEP vs. LEP) or age-specific (<30y vs. >55y) contrast terms.

For Exploratory Data Analysis (EDA) and visualization of gene expression data, normalized data were corrected for batch effects using ComBat [54] – an empirical Bayes approach for adjusting data for batch effects that is robust to outliers in small sample sizes as applied in the Bioconductor sva package [55], with no covariates included in the model for batch adjustment [56]. After batch adjustment, expression data were pre-filtered as described above using the detection p-values. PCA and hierarchical clustering were used pre- and post-ComBat treatment for visualization to illustrate removal of batch effects and the clustering of reference sample. All analyses using hierarchical clustering of samples were done using complete linkage of Euclidean distances in the pvclust package which calculates Approximately Unbiased (AU) p-values and Boostrap Probability (BP) values for each cluster [57].

For Exploratory Data Analysis (EDA) and visualization of DNA methylation data, methylation beta-values – which have a more direct biological interpretation, were plotted across CpG sites ordered by their chromosomal mapping. Beta values range from 0 to 1 and denote hypo- (β-val < 0.25), hemi- (0.25 < β-val < 0.75) and hyper-methylated (β-val > 0.75) methylation levels. Differential methylation was annotated based on DM analysis of methylation M-values.

## SUPPLEMENTARY MATERIAL FIGURES AND TABLES


