# Supplementary material for: Age-related gene expression in luminal epithelial cells is driven by a microenvironment made from myoepithelial cells
Source: Aging (Albany NY). 2017 Oct 9;9(10):2026–51. doi: 10.18632/aging.101298 (PMC5680554; doi:10.18632/aging.101298)
Supplement: Supplementary file 1 [file aging-09-2026-s001.pdf]

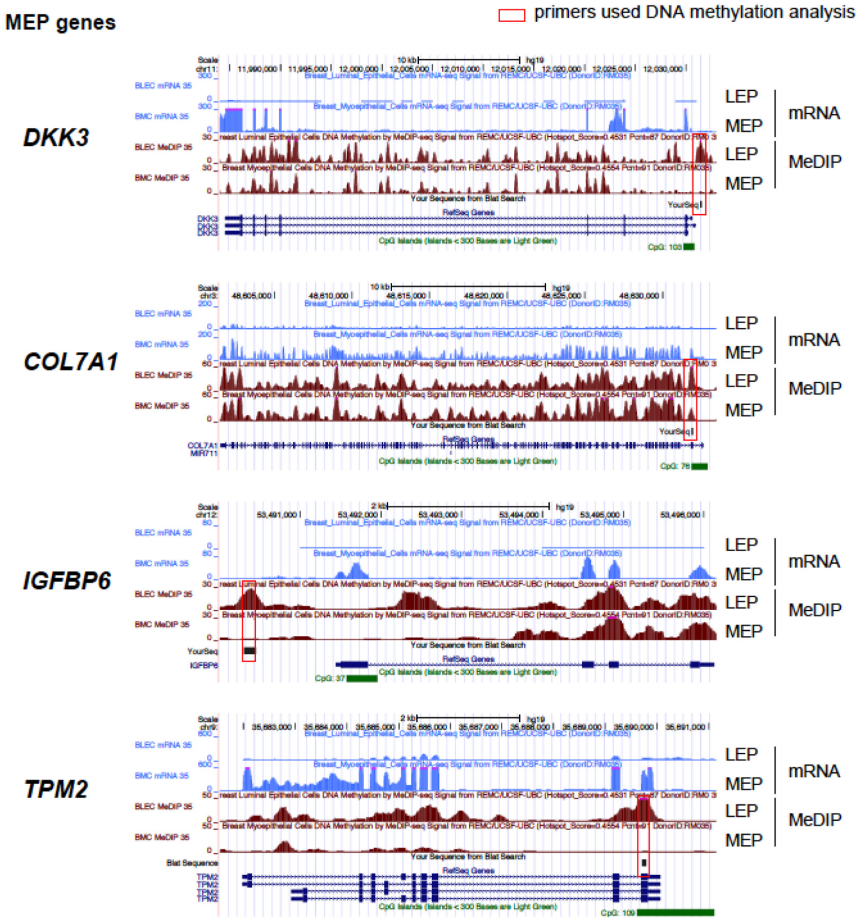

Figure S1. Lineage specific gene expression and DNA methylation states in 4th passage HMECs are comparable to those from Roadmap epigenomics data. Comparison of gene expression and DNA methylation states in lineage-specifix gene probes used in in this study between 4th passage HMECs and Roadmap epigenomics data. Genomic maps with Roadmap epigenomics data were available from UCSC Genome Browser. RNA-seq and MeDIP data were used to show gene expression (mRNA) shown in light blue and DNA methylation shown in Roadmap data. Red rectangular boxes indicated that the regions used DNA methylation analysis qPCR in this study. Green boxes shown CpG islands.

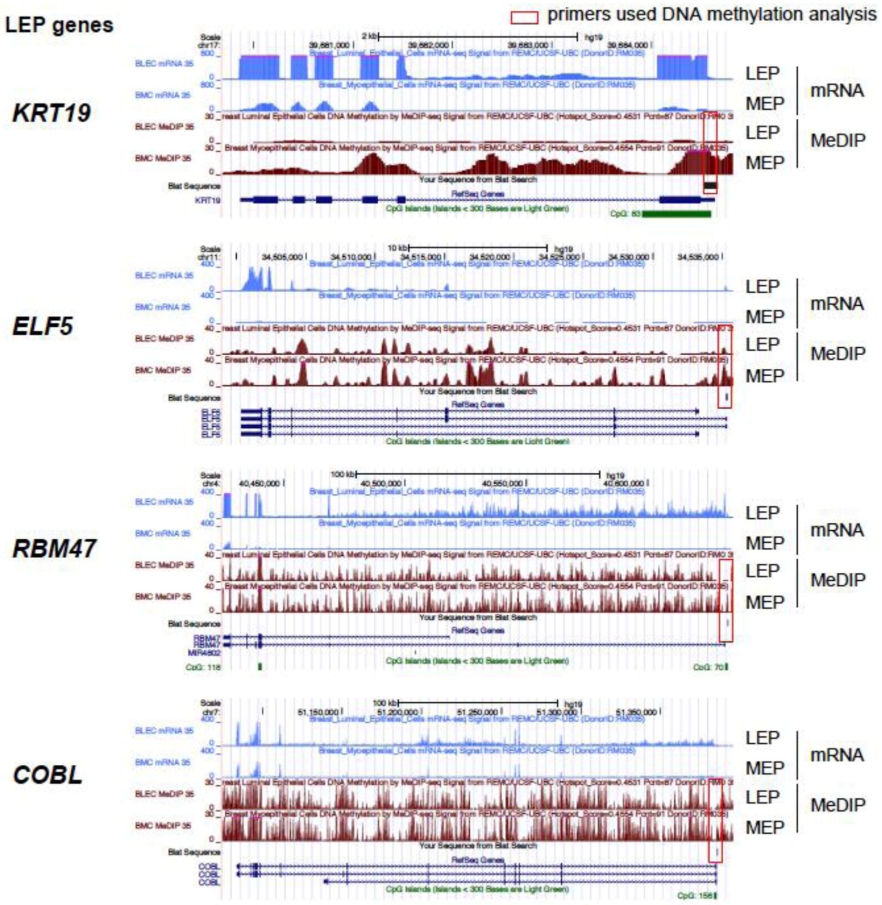

Figure S1. (cont.)

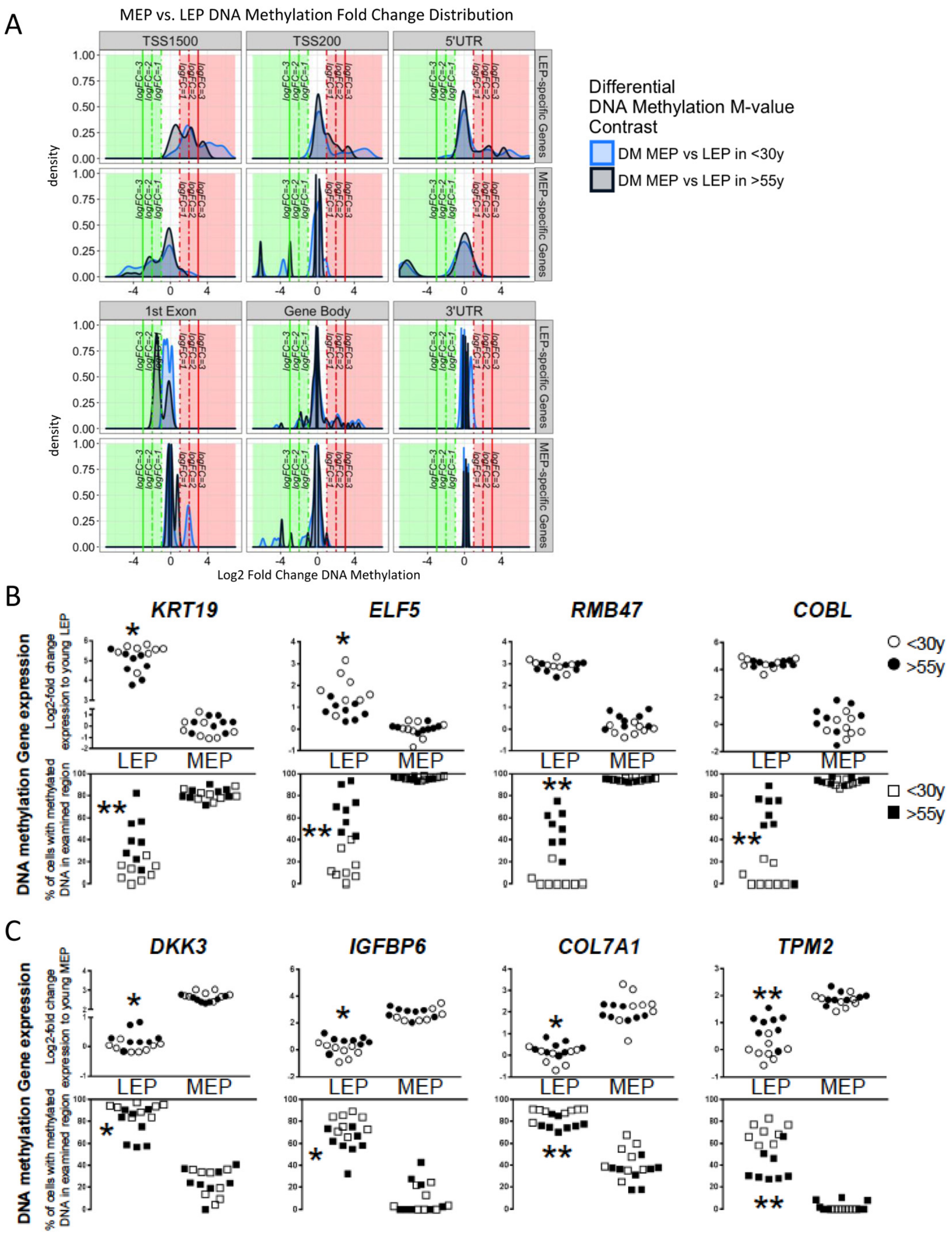

Figure S2. Detection of age-dependent differentiation methyation in the the lineage specific genes in LEP and MEP. Infinium 450K methylation arrays were used to evaluate differential methylation (DM) based of M-values of lineage-specific genes represented by the probeset at 247 CpG sites. (**A**) Kernel Density Estimates (KDE) of distributions of log2 fold changes (LFC) between MEP vs. LEP DNA methylation in <30y (light blue) and >55y (dark blue) subjects for LEP-specific (top panel) and MEP-specific (bottom panel). Colored regions and lines highlight fraction of genes which show lineage-specific differential methylation: ≥ 1-, ≥ 2-, ≥ 3- log2 fold change and Benjamini-Hochberg (BH) adj. p-val < 0.05, < 0.01, < 0.001, with negative LFC values (green area) indicating higher methylation in LEP and positive values (red area) higher methylation in MEP. KDE are faceted by annotated locations of CpG sites respective to gene regions: TSS1500, TSS200, 5'UTR, 1st Exon, Gene Body and 3'UTR. Quantitative PCR of McrBC using (**B**) Luminal- and (**C**) myoepithelial-specific probe sets were used to identify age-dependent changes in lineage-specific gene expression and and DNA methylation patterns in FACS enriched LEP (n=16) and in MEP (n=16) from early passage HMEC strains. Age-dependent lineage-specific changes were validated using two approaches. Dysregulation of lineage specific gene expression with age in LEP was associated with age-dependent DNA methylation patterns. * and ** showed in all figures indicates statistical significances at p<0.05 and p<0.01, respectively.

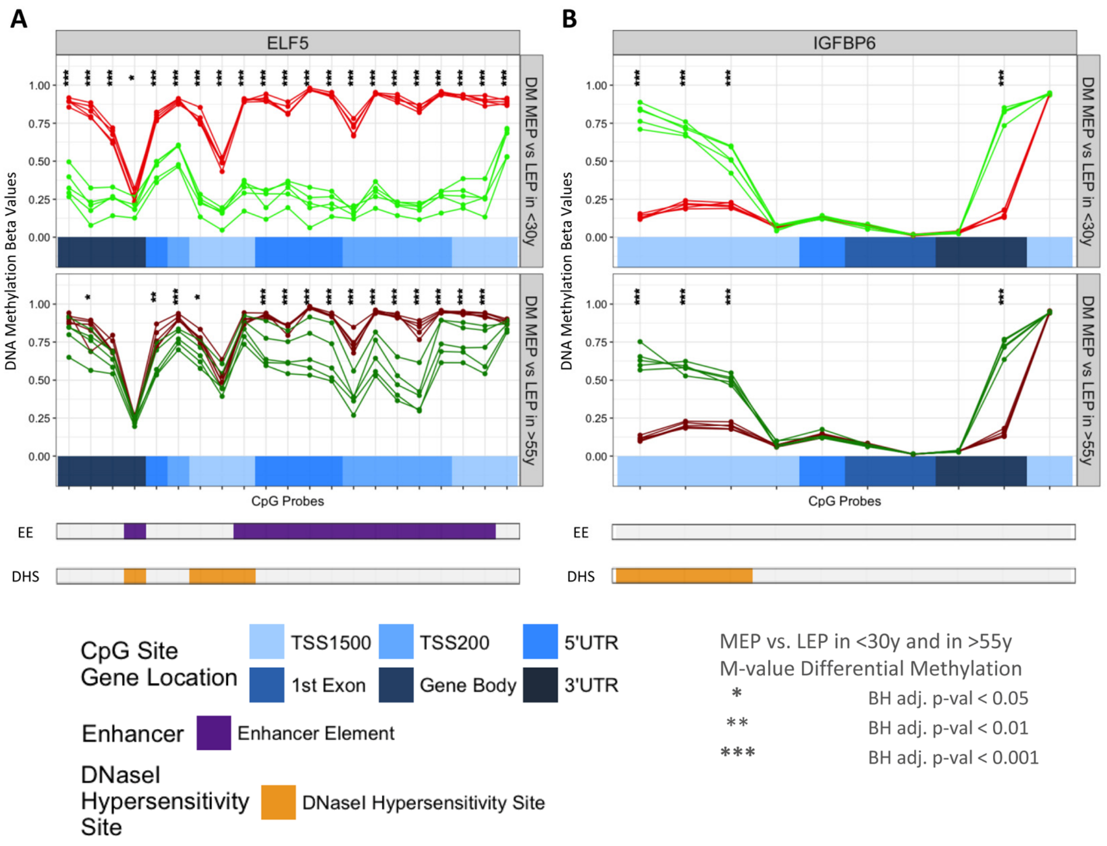

Figure S3. Lineage-dependent differential methylation across the regulatory regions and gene bodies of ELF5 and IGFBP6. DNA methylation beta-values across ELF5 and IGFBP6 CpG sites for <30y LEP (green) and <30y MEP (red), and >55y LEP (dark green) and >55y MEP (dark red) are plotted and range from 0-1 denoting hypo- (β-val < 0.25), hemi- (0.25 < β-val < 0.75) and hyper-methylated (β-val > 0.75) methylation levels. Corresponding annotated locations of CpG sites respective to gene regions: TSS1500, TSS200, 5'UTR, 1st Exon, Gene Body and 3'UTR (shades of blue), as well as annotated Enhancer Element regions (purple) and DNaseI Hypersensitivity Sites (orange) are shown on tracks below. Significance of lineage-specific differential methylation (DM) based on corresponding M-values between MEP and LEP in <30y (top panel) and >55y (bottom panel) are denoted by asterisks: (*) Benjamini-Hochberg (BH) adj. p-val < 0.05, (**) < 0.01, (***) < 0.001. Loss of lineage-specific methylation with age is indicated by loss of corresponding asterisks between top and bottom panel along each CpG probe track.

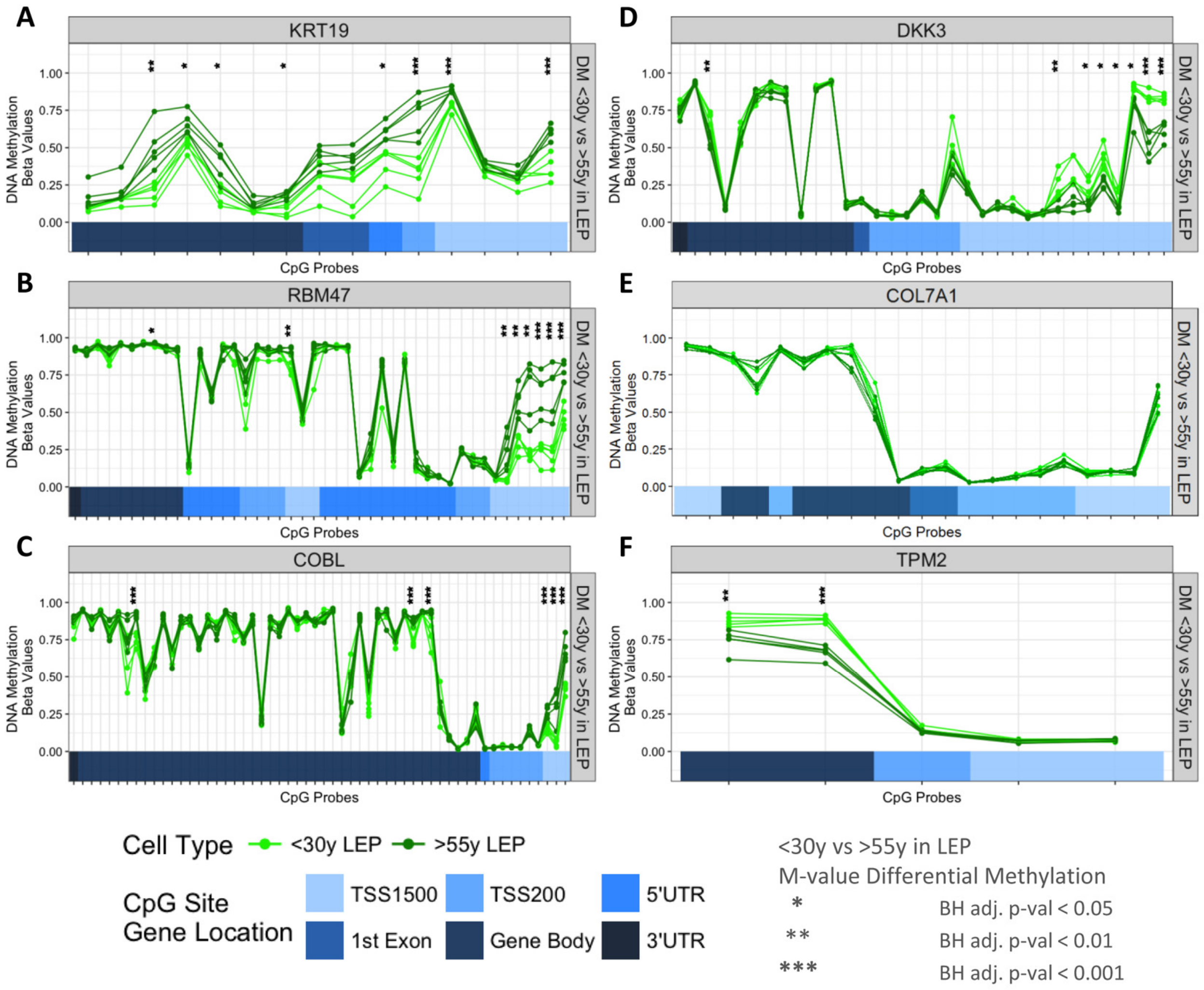

Figure S4. Age-dependent differential methylation across the regulatory regions and gene bodies of the probeset genes in LEP. DNA methylation beta-values across CpG sites in (**A**) KRT19, (**B**) RBM47, (**C**) COBL, (**D**) DKK3, (**E**) COL7A, and (**F**) TPM2 for <30y LEP (green) and >55y LEP (dark green) are plotted and range from 0-1 denoting hypo- (β-val < 0.25), hemi- (0.25 < β-val < 0.75) and hyper-methylated (β-val > 0.75) methylation levels. Corresponding annotated locations of CpG sites respective to gene regions: TSS1500, TSS200, 5'UTR, 1st Exon, Gene Body and 3'UTR (shades of blue). Significance of age-specific differential methylation (DM) based on corresponding M-values between <30y and >55y LEP are denoted by asterisks: (*) Benjamini-Hochberg (BH) adj. p-val < 0.05, (**) < 0.01, (***) < 0.001.

Table S1. Sample list of organoids and pre-stasis 4th passage HMECs.

Organoids				4th passage			
<30y		>55y		<30y		>55y	
Strain	Age	Strain	Age	Strain	Age	Strain	Age
160	16y	112R	61y	160	16y	117R	56y
53R	19y	96L	62y	48R	16y	191L	56y
59L	23y	71C	65y	240L	19y	153L	60y
		122R	66y	356E	21y	112R	61y
				59L	23y	71C	65y
				51L	28y	122L	66y
				172L	28y	29	68y
				124	29y	429ER	72y

Table S2. qPCR primer sequences.

Primers for gene expression

Gene	Sequence (5'-3')
GAPDH	AAGGTGAAGGTCGGAGTCAAC
	GGGGTCATTGATGGCAACAATA
RPS18	GGGCGGCGGAAAATAG
	CGCCCTCTTGGTGAGGT
TBP	GAGCTGTGATGTGAAGTTTCC
	TCTGGGTTTGATCATTCTGTAG
TP63	TGCTGTTGCCTGTACGTTTC
	ACGAAGATCCCCAGATGATG
DKK3	TGGGGAAATGTGGAGAAGAG
	TCATCTGCAACAGCTGAAGG
COL7A1	AATTCTCCATGTGGCTGACC
	TGATCAGGATGCAGACCTTG
IGFBP6	TGTGACCATCGAGGCTTCTAC
	TTCCATTGCCATCTGGAGAC
TPM2	AAGAAGCTGAAGGGGACAGAG
	AGGCCACATCTGCCTCAG

PROM1	TCAGATCTGTGAACGCCTTG
	GTCGGAAACTGGCAGATAGC
KRT19	AACGGCGAGCTAGAGGTGA
	GGATGGTCGTGTAGTAGTGGC
ELF5	TAGGGAACAAGGAATTTTTCGGG
	GTACACTAACCTTCGGTCAACC
RBM47	GGCATTAAGGGTTGATGGTG
	GAAGTGCGGCAAGTCTTTTC
COBL	AAGGCAAGCCTTGATGGAC
	TGGCCTCTGTTCATTCACAC

Primers for DNA methylation

Gene	Sequence (5'-3')
TIMP3	TGTAATTCCCACCCCTCTTG
	GTTGGCCTTTCAGCAAGTTC
CDX1	GGGTTTCCCCCTTTGATTC
	CACCCAGGCCTTTTATAGCTC
BCLAF1	CTGGCTGCTATTAAGATGTTGC
	TGACAAAACACCCACCCTAC
DKK3	AGCTCTGCTCCTTCCTAACTTC
	TGGCCTGATCGTCTAACTTCTC
COL7A1	ACTGGCTGCTCCAGAGAAAG
	CTTTACGCCGCTGACATTG
IGFBP6	ATCCCTCTTCCTCTCTCTTGTG
	AGGGACTACTCAGCATCTTTGC
TMP2	GGTCCTCAGCTTGCTTCTTG
	ATGCTGAAGCTGGACAAGG
KRT19	AACCCTGGTCTCAGAAGCTG
	TCTCAGGAGCCTGCAAATTC
ELF5	GCGTGCAGTGGAAATAAAGAC
	CACACTGTATGTCACCGTCATC
RBM47	TCCCAAGAAACCCAGATGTC
	CTTAGCGCTCCACTGAAATG
COBL	GTTTGCCAACCTGATTCACTG
	GAGGTGAAGTTGGGCAGATAAG
